# South African lamb and cardiovascular disease risk

**Published:** 2007-07

**Authors:** R Delport, HC Schönfeldt

**Affiliations:** Department of Chemical Pathology, Faculty of Health Sciences, University of Pretoria; School of Agriculture and Food Sciences, Faculty of Natural and Agricultural Sciences, University of Pretoria

Recent analyses of the composition of South African lamb have yielded more positive findings than those reported previously. Evidently the fat content is much less than previously reported, and more appropriate according to recommendations set for the prevention of cardiovascular disease. The protein content and the vitamin constituents are proposed to be conducive to the metabolism of methionine, thereby preventing accumulation of homocysteine in the circulation with lamb intake.

This editorial evaluates the relevance of these results in terms of the possible implications for cardiovascular disease.

## The composition of South African lamb

New data on South African lamb shows that it contains on average 42% less fat and 59% less energy (kJ)^1^ than previously reported by the Medical Research Council.^2^ Previous data were compiled from international reports. Table 1 depicts the new findings.

**Table 1:** Nutrient Content Of Cooked Loin Of SA Lamb (Per 100 g)

*Nutrient*	*Units*	*Cooked lamb loin cut*
Proximate analysis		
Energy	kJ	755
Protein	g	27.8
Fat	g	7.8
Moisture	g	63.5
Fatty acids		
Saturated fatty acids (SFA)	g	3.53
Monounsaturated fatty acids (MUFA)	g	3.05
Polyunsaturated fatty acids (PUFA)	g	0.24

The study of van Heerden *et al.* 1 reports that South African lamb and mutton contains on average less than 10% fat, with the shoulder cut containing 9.86 g, the loin containing 7.80 g and the leg containing 7.67 g fat. According to recommendations of both the Heart Foundation (www.heartfoundation.com) and the Cancer Foundation (www.cancer.org), meat should contain less than 10% fat, and intake of South African lamb and mutton are therefore acceptable. Furthermore, it is important to note that the nutrient content of leaner meat is higher, as fat dilutes the nutrients in a protein matrix.

Ruminant meat is a natural source of conjugated linoleic acid (CLA)^3^,^4^ and of the different ruminants, lamb is the richest meat source of CLA.^5^ Lamb is also reported to have a higher proportion of unsaturated fatty acids compared with saturated fatty acids (SFAs),^5^ which is favourable as increased risk of cardiovascular disease is related to the intake of diets high in saturated fatty acids.^6^ The proportion of fatty acids will be affected by trimming as lean meat is higher in polyunsaturated fatty acids (PUFA) and lower in SFAs than untrimmed meat.^4^ Lean red meat is a source of polyunsaturated fats, including omega-3 fatty acid, and pasture feeding contributes significantly to long-chain omega-3 fatty acid intake in the diet.^4^ In contrast, meat from grain-fed animals has no omega-3 fatty acids as grain does not contain omega-3 fatty acids, but is high in omega-6 fatty acids (linoleic acid).^6^

Cooked loin of South African lamb appears to be an excellent source of protein as reported in the study of van Heerden *et al.*^1^ Furthermore, the protein contains all eight essential amino acids^6^-^8^ and is labelled as a source of haem-iron, B vitamins and zinc.^9^ This study once again confirms findings of the high mineral and vitamin content of lamb and mutton, as depicted in Table 2. Folate concentrations were not determined in this study but previous reports showed that lean red meat is also a major source of bio-available folate.^10^

**Table 2:** Nutrient Density Of Selected Micronutrients And Vitamins In Cooked Loin Of SA Lamb

*Micronutrients*	Nutrient density*
Minerals	
Zinc	3.18
Iron	1.23
Magnesium	0.91
Vitamins	
Vitamin B_6_	1.59
Vitamin B_12_	1.32
Niacin	1.24

*Nutrient density values > 1 indicate that the sample is a good source of the specific micronutrient.

## Concern about meat intake and elevated homocysteine levels

Whether elevated homocysteine is causally related to CVD is still uncertain. What is known is that gender, age, folate intake, smoking status and coffee consumption have been identified as the strongest determinants of total homocysteine concentration in the general population,^11^ folate intake and coffee consumption being modifiable determinants. The most important non-modifiable determinant is the 677 C>T polymorphism in the gene that encodes 5,10-methylene-tetrahydrofolate reductase (MTHFR),^11^ an enzyme that irreversibly reduces 5,10-methylene-THF to 5-methyl-THF in the remethylation pathway from homocysteine to methionine.^11^

Other factors that influence tHcy levels in the general population include blood levels of folate, vitamin B_12_ and betaine, as well as renal function.^12^-^16^ It may be a significant observation that high meat intake is not reported to be associated with hyperhomocysteinemia, whereas the vegan diet is.^17^,^19^ An inverse association was observed between meat intake and plasma homocysteine concentration, while vitamin B12 concentration was observed to decrease progressively.^20^ Dietary methionine intake appears to have no observable effect on plasma homocysteine concentration.^20^,^21^

In habitual diets where folate intake is adequate, lowered vitamin B_12_ intake from animal foods leads to depleted plasma vitamin B_12_ concentrations, with a concomitant increase in homocysteine concentrations.^20^ A high-protein diet did, however, increase circulating homocysteine levels to a greater extent throughout the day.^21^ This may be of some concern as higher postprandial homocysteine levels were observed to be related to cardiovascular disease risk, independent of fasting levels.^22^ A study by Verhoef, *et al.*, however, reports markedly lower increases in circulating levels of homocysteine following ingestion of dietary methionine compared with ingestion of free methionine, with serine and cystine attenuating the homocysteine-raising effect of free methionine.^23^

Moderately elevated homocysteine levels relate to higher risk of cardiovascular disease, as is evident from epidemiological reviews.^24^,^25^ Lowering homocysteine with dietary folate and B vitamins in populations at high risk for CVD has, however, failed to show treatment benefits.^26^-^28^ The latest large prospective study performed in women reports marked attenuation of the association of homocysteine with CVD after adjusting for risk factors. Furthermore, neither the MTHFR 677C>T genotype or dietary intake of folate and B vitamins appeared to mediate or modify the association of homocysteine with CVD in healthy women.^29^

Concern about methionine intake resulting in elevated homocysteine levels is possibly allayed by the fact that homocysteine has not yet inconclusively been established as a CVD risk factor. Moderate intake of meat will certainly not result in elevated fasting homocysteine levels over time, provided that folate intake is adequate. It is a remarkable phenomenon that meat, and especially lamb, contains methionine as well as other amino acids such as cysteine and serine, which evidently favours its metabolism and attenuates high postprandial homocysteine levels. The concomitant intake of methionine, co-substrate (folate) and co-factors (vitamin B_12_ and vitamin B_6_) will in all probability enhance the effective transmethylation and transsulfuration of homocysteine, thereby preventing accumulation of homocysteine over time.

To conclude, South African lamb may be considered favourably for dietary intake of proteins, certain micronutrients and vitamins if consumed in moderation.

## Key message

● South African lamb is recommended as a dietary source of protein as it contains less fat than previously reported.
● Moderate intake of lamb is not envisaged to affect circulating homocysteine levels adversely.
